# Lung disease recalling paraseptal emphysema in a patient with Goltz syndrome

**DOI:** 10.1186/s40248-016-0069-9

**Published:** 2016-09-13

**Authors:** Rosaria Cortese, Salvatore Savasta, Silvia Di Stasi, Tiziana Boggini, Chiara Trabatti, Roberto Dore, Giulia Maria Stella

**Affiliations:** 1 Cardiothoracic Department, Section of Pneumology, IRCCS Policlinico San Matteo, 27100 Pavia, Italy; 2 Department of Pediatrics, IRCCS Policlinico San Matteo, 27100 Pavia, Italy; 3 Unit of Radiology, IRCCS Policlinico San Matteo, 27100 Pavia, Italy

**Keywords:** Emphysema, Goltz syndrome, Lung disease

## Abstract

**Background:**

Goltz syndrome is a rare, genetic disorder mainly occurring in female patients.

**Case presentation:**

The case presented here is, to the best of our knowledge, the first description of the occurrence of lung parenchymal alterations in a young female patient affected by Goltz syndrome. Although pulmonary involvement is not known in patients affected by X-linked Goltz syndrome, the case here described is related to the even rarer autosomal form of the disease, as in this case. It is thus conceivable that in such different genetic setting the involvement of lung parenchyma may be unveiled through atypical emphysematous lesions.

**Conclusion:**

This report suggested - for the first time time - a rationale for a lung function and imaging screening in patients affected by Goltz syndrome at least in its autosomal form.

## Background

Pulmonary emphysema (PE) defines an abnormal, permanent enlargement of the airspaces distal to the terminal bronchioles, accompanied by destruction of their walls. Most often PE is related to chronic obstructive pulmonary disease (COPD) or alpha_1_-antitrypsin deficiency and it is associated to cigarette smoking [1]. High-resolution computed tomography (HRCT) scanning identifies an especially reliable noninvasive tool for demonstrating the pathology of emphysema; notably high correlation with histopathology has been shown [2]. Disease progression leads to inadequate oxygenation and respiratory failure, disability, cachexia ultimately responsible of patient’s death; lung transplantation may provide the only effective remedy in advanced stages [3]. PE is usually classified into the following three main subtypes: i) centrilobular emphysema (CLE), the most common morphological subtype in which the pathological process begins near the centre of the secondary pulmonary lobule in the region of the proximal respiratory bronchiole; ii) panlobular emphysema (PLE) defined by permanent destruction of the entire acinus distal to the respiratory bronchioles; iii) paraseptal emphysema (PSE). The latter defines emphysematous lesions caused by selective destruction of the distal acinus; most often the term paraseptal is used to describe parenchymal lesions located near the pleural surface close to the chest wall and in the interlobar fissures. Notably PSE is rarely associated with significant symptoms or physiologic impairment [4, 5].

The case presented is worth to be reported since it is - to the best of our knowledge - the first description of the occurrence of lung parenchymal alterations in a young female patient affected by Goltz syndrome. The latter, also named as focal dermal hypoplasia (FDH), defines a rare, genetic disorder, with fewer than 300 cases reported, most of whom are female. It was first described by Robert Goltz in 1962 and it is characterized by distinctive skin abnormalities (atrophy and linear pigmentation) and a wide variety of defects affecting eyes, teeth, as well as skeletal, urinary, gastrointestinal, cardiovascular, and central nervous systems [6]. In addition, herniation of fat through dermal defects, multiple papillomas of the mucous membranes and the skin as well as digital defects as syndactyly, polydactyly or campodactyly may frequently occur. Mental retardation affects some patients. Striated bones may be detected [7]. FDH is mainly inherited in an X-linked dominant fashion, with in utero lethality in males. In the vast majority of cases the syndrome is caused by lesions affecting the *PORCN* gene located on the X chromosome (Xp11.23 [8]). *PORCN* encodes for the human homolog of Drosophila melanogaster porcupine, an endoplasmic reticulum protein involved in secretion of Wnt proteins [9]. Moreover, the chromosomal region 9q32 has been suggested as putative locus for an autosomal Goltz syndrome form, as in the case here discussed [10].

## Case presentation

**Fig. 1:**
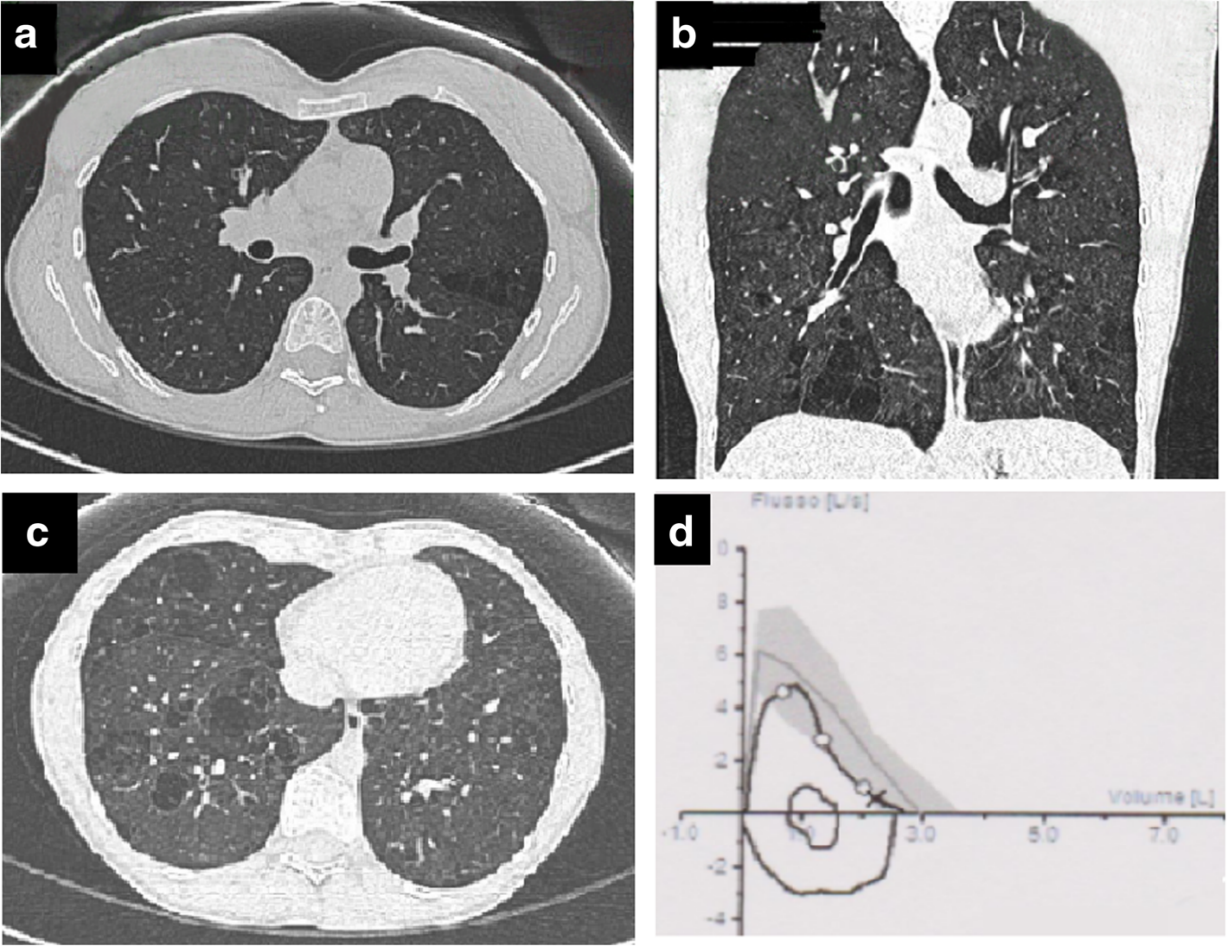
Panel **a**, **b**, **c**: HRCT scan images demonstrating atypical paraseptal emphysema; Panel **d**: flow-volume loops showing normal values

A 36-year-old woman affected by Goltz syndrome was admitted to our observation to evaluate unclear alteration of the lung basis suggesting bullous dystrophy and occasionally reported during a CT scan, performed to study an abdominal pain subsequently spontaneously solved. Goltz syndrome was diagnosed and reported [10] when the patient was 6 years old. The clinical history was essentially characterized by a number of not complicated bone fractures in absence of multi-organ abnormalities. She presented with some facial dysmorphic signs and showed linear cutaneous aplasia on her face. With respect to the respiratory system the patient referred a silent history. She was an occasional smoker. She managed a dry cleaner’s, without direct exposure to toxic cleansers and vapors. She did not refer any thoracic/respiratory problem either in the childhood or in the more recent years so that no previous chest radiograms were available. At the onset, we performed a chest HRCT scan which documented emphysematous destruction with aspects that partially recalled PE featuring an unusual distribution pattern. In detail, the CT scan allowed to report in many sections a single chaplet layer of bubbles with thin walls formed by interlobular septa typical for PE (Fig. 1,a). As opposite, it differed from typical PE because lesions were not entirely peripheral and interested mainly the lobules localized at the center of the lung (Fig 1,b). Moreover, chest alterations were consistent neither with CLE, since they mainly affected lower lobes instead of the expected upper ones (Fig. 1,c), nor with PLE because of the presence of cyst-like lesions. The latter cannot properly be defined as cysts due to the absence of thick walls and the presence of septa and vessels in their middle. The global spirometry showed normal lung function together with a slightly reduced diffusing capacity (DL_CO_) (Fig. 1,d). In order to exclude a genetic origin of the emphysema related to alpha-_1_ antitrypsin (A1AT) deficiency we performed isoelectric focusing and a MM genotype (associated to the production of a normal level of enzyme) was demonstrated. Notably, seric levels of A1AT were higher than normal and RCP was slightly increased too as coherent with an inflammatory condition. Bronchoscopy allowed us to exclude a tracheal diverticulum as suspected at the CT scan. The patient continued the follow up, under clinical and instrumental surveillance.

## Conclusion

Overall the above reported findings allow some relevant considerations. Although pulmonary involvement has never been reported in patients affected by X-linked Goltz syndrome, the case here described is related to the even rarer autosomal form of the disease, featuring lesions in the 9q32 chromosomal focus. It is, thus, conceivable that in such different genetic setting the involvement of lung parenchyma may be unveiled through atypical emphysematous lesions. This aspect can be more intriguing if considered that a genetic linkage of a gene for the TSC complex 1 to loci in 9q32-9q34 is well known [11] and that pulmonary lymphangioleiomyomatosis can develop in the lungs of patients affected by tuberous sclerosis [12]. On this perspective it could be hypothesized that the specific genetic asset may be associated with the onset of an emphysematous phenotype characterized by cyst-like lesions. This report suggests - for the first time - a rationale for a lung function and imaging screening in patients affected by Goltz syndrome at least in its autosomal form.

## Abbreviations

A1AT, alpha-_1_ antitrypsin; CLE, centrilobular emphysema; COPD, chronic obstructive pulmonary disease; FDH, focal dermal hypoplasia; HRCT, high resolution computed tomography; PE, pulmonary emphysema; PLE, panlobular emphysema; PSE, paraseptal emphysema
